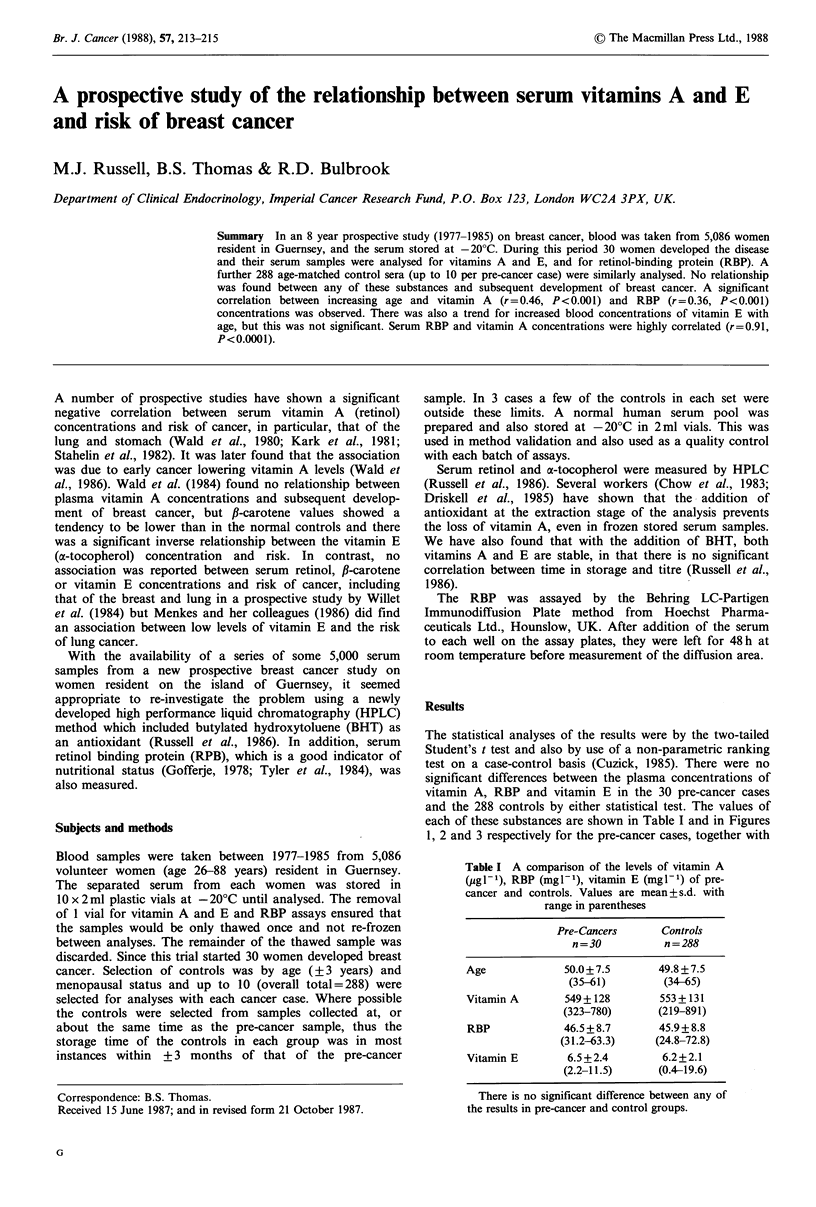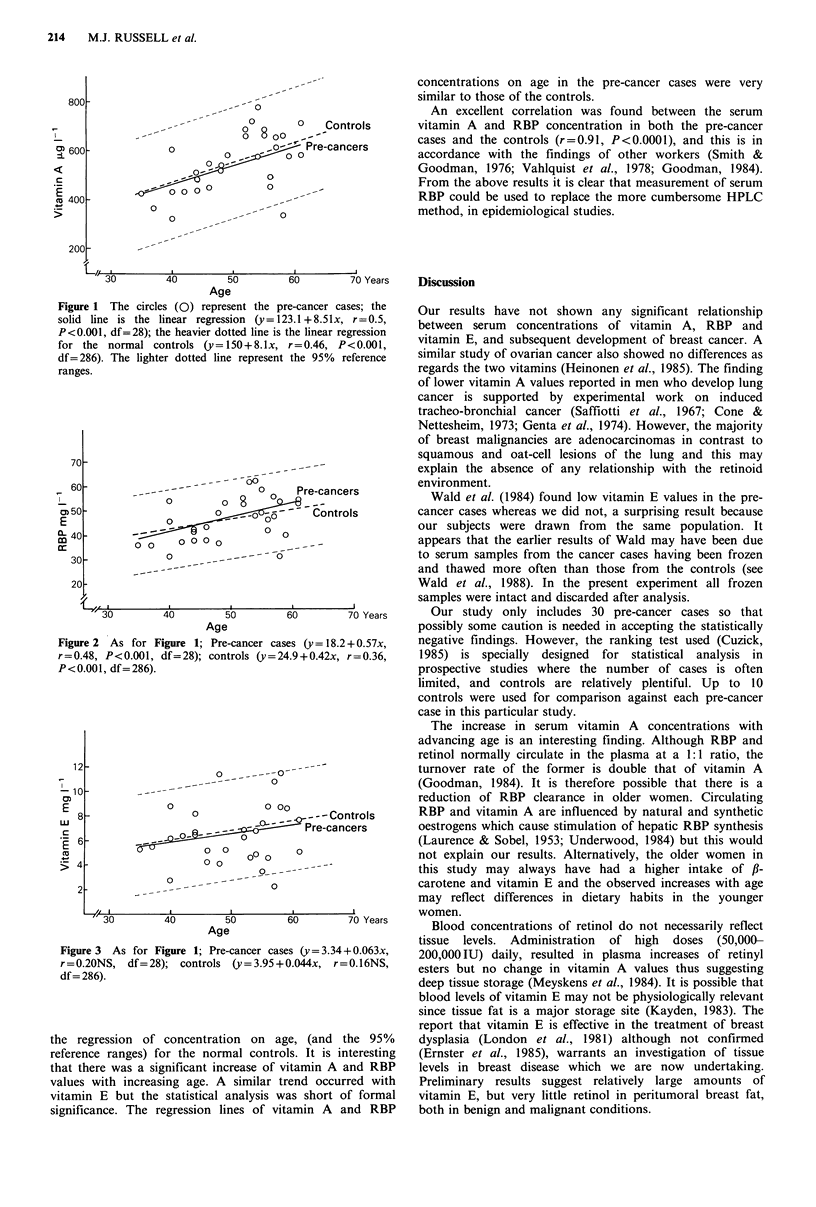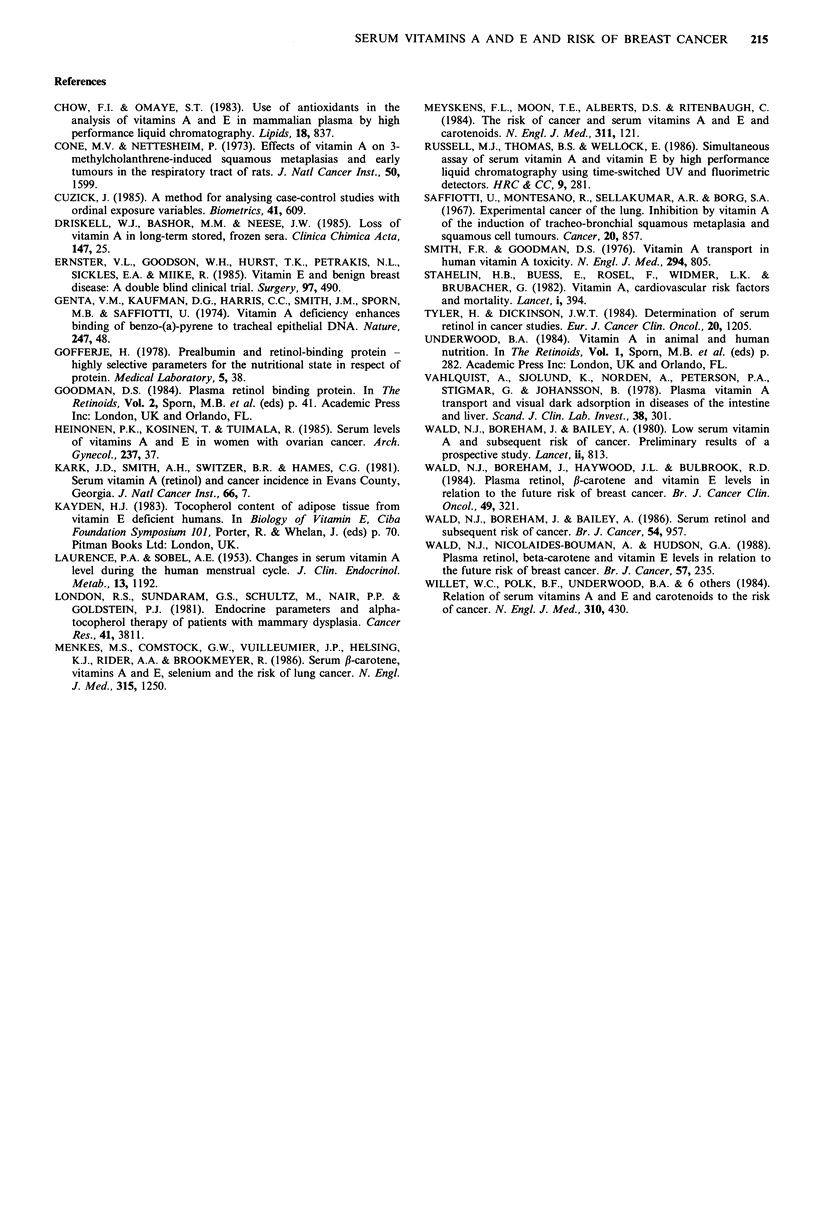# A prospective study of the relationship between serum vitamins A and E and risk of breast cancer.

**DOI:** 10.1038/bjc.1988.45

**Published:** 1988-02

**Authors:** M. J. Russell, B. S. Thomas, R. D. Bulbrook

**Affiliations:** Department of Clinical Endocrinology, Imperial Cancer Research Fund, London, UK.

## Abstract

In an 8 year prospective study (1977-1985) on breast cancer, blood was taken from 5,086 women resident in Guernsey, and the serum stored at -20 degrees C. During this period 30 women developed the disease and their serum samples were analysed for vitamins A and E, and for retinol-binding protein (RBP). A further 288 age-matched control sera (up to 10 per pre-cancer case) were similarly analysed. No relationship was found between any of these substances and subsequent development of breast cancer. A significant correlation between increasing age and vitamin A (r = 0.46, P less than 0.001) and RBP (r = 0.36, P less than 0.001) concentrations was observed. There was also a trend for increased blood concentrations of vitamin E with age, but this was not significant. Serum RBP and vitamin A concentrations were highly correlated (r = 0.91, P less than 0.0001).


*Br. J. Cancer* (1988), **57**, 213–215 © The Macmillan Press Ltd., 1988

### A prospective study of the relationship between serum vitamins A and E and risk of breast cancer

M.J. Russell, B.S. Thomas & R.D. Bulbrook

*Department of Clinical Endocrinology, Imperial Cancer Research Fund, P.O. Box 123, London WC2A 3PX, UK.*

**Summary** In an 8 year prospective study (1977–1985) on breast cancer, blood was taken from 5,086 women resident in Guernsey, and the serum stored at −20°C. During this period 30 women developed the disease and their serum samples were analysed for vitamins A and E, and for retinol-binding protein (RBP). A further 288 age-matched control sera (up to 10 per pre-cancer case) were similarly analysed. No relationship was found between any of these substances and subsequent development of breast cancer. A significant correlation between increasing age and vitamin A ($r=0.46$, $P<0.001$) and RBP ($r=0.36$, $P<0.001$) concentrations was observed. There was also a trend for increased blood concentrations of vitamin E with age, but this was not significant. Serum RBP and vitamin A concentrations were highly correlated ($r=0.91$, $P<0.0001$).

A number of prospective studies have shown a significant negative correlation between serum vitamin A (retinol) concentrations and risk of cancer, in particular, that of the lung and stomach (Wald *et al.*, 1980; Kark *et al.*, 1981; Stahelin *et al.*, 1982). It was later found that the association was due to early cancer lowering vitamin A levels (Wald *et al.*, 1986). Wald *et al.* (1984) found no relationship between plasma vitamin A concentrations and subsequent development of breast cancer, but β-carotene values showed a tendency to be lower than in the normal controls and there was a significant inverse relationship between the vitamin E (α-tocopherol) concentration and risk. In contrast, no association was reported between serum retinol, β-carotene or vitamin E concentrations and risk of cancer, including that of the breast and lung in a prospective study by Willet *et al.* (1984) but Menkes and her colleagues (1986) did find an association between low levels of vitamin E and the risk of lung cancer.

With the availability of a series of some 5,000 serum samples from a new prospective breast cancer study on women resident on the island of Guernsey, it seemed appropriate to re-investigate the problem using a newly developed high performance liquid chromatography (HPLC) method which included butylated hydroxytoluene (BHT) as an antioxidant (Russell *et al.*, 1986). In addition, serum retinol binding protein (RPB), which is a good indicator of nutritional status (Gofferje, 1978; Tyler *et al.*, 1984), was also measured.

### Subjects and methods

Blood samples were taken between 1977–1985 from 5,086 volunteer women (age 26–88 years) resident in Guernsey. The separated serum from each women was stored in 10 × 2 ml plastic vials at −20°C until analysed. The removal of 1 vial for vitamin A and E and RBP assays ensured that the samples would be only thawed once and not re-frozen between analyses. The remainder of the thawed sample was discarded. Since this trial started 30 women developed breast cancer. Selection of controls was by age (±3 years) and menopausal status and up to 10 (overall total = 288) were selected for analyses with each cancer case. Where possible the controls were selected from samples collected at, or about the same time as the pre-cancer sample, thus the storage time of the controls in each group was in most instances within ±3 months of that of the pre-cancer sample. In 3 cases a few of the controls in each set were outside these limits. A normal human serum pool was prepared and also stored at −20°C in 2 ml vials. This was used in method validation and also used as a quality control with each batch of assays.

Serum retinol and α-tocopherol were measured by HPLC (Russell *et al.*, 1986). Several workers (Chow *et al.*, 1983; Driskell *et al.*, 1985) have shown that the addition of antioxidant at the extraction stage of the analysis prevents the loss of vitamin A, even in frozen stored serum samples. We have also found that with the addition of BHT, both vitamins A and E are stable, in that there is no significant correlation between time in storage and titre (Russell *et al.*, 1986).

The RBP was assayed by the Behring LC-Partigen Immunodiffusion Plate method from Hoechst Pharmaceuticals Ltd., Hounslow, UK. After addition of the serum to each well on the assay plates, they were left for 48 h at room temperature before measurement of the diffusion area.

### Results

The statistical analyses of the results were by the two-tailed Student's $t$ test and also by use of a non-parametric ranking test on a case-control basis (Cuzick, 1985). There were no significant differences between the plasma concentrations of vitamin A, RBP and vitamin E in the 30 pre-cancer cases and the 288 controls by either statistical test. The values of each of these substances are shown in Table I and in Figures 1, 2 and 3 respectively for the pre-cancer cases, together with

**Table I** A comparison of the levels of vitamin A ($\mu g\,l^{-1}$), RBP ($mg\,l^{-1}$), vitamin E ($mg\,l^{-1}$) of pre-cancer and controls. Values are mean ± s.d. with range in parentheses

| | *Pre-Cancers* $n=30$ | *Controls* $n=288$ |
|---|---|---|
| Age | 50.0 ± 7.5 (35–61) | 49.8 ± 7.5 (34–65) |
| Vitamin A | 549 ± 128 (323–780) | 553 ± 131 (219–891) |
| RBP | 46.5 ± 8.7 (31.2–63.3) | 45.9 ± 8.8 (24.8–72.8) |
| Vitamin E | 6.5 ± 2.4 (2.2–11.5) | 6.2 ± 2.1 (0.4–19.6) |

There is no significant difference between any of the results in pre-cancer and control groups.

Correspondence: B.S. Thomas.
Received 15 June 1987; and in revised form 21 October 1987.

**Figure 1** The circles (○) represent the pre-cancer cases; the solid line is the linear regression ($y=123.1+8.51x$, $r=0.5$, $P<0.001$, df=28); the heavier dotted line is the linear regression for the normal controls ($y=150+8.1x$, $r=0.46$, $P<0.001$, df=286). The lighter dotted line represent the 95% reference ranges.

**Figure 2** As for **Figure 1**; Pre-cancer cases ($y=18.2+0.57x$, $r=0.48$, $P<0.001$, df=28); controls ($y=24.9+0.42x$, $r=0.36$, $P<0.001$, df=286).

**Figure 3** As for **Figure 1**; Pre-cancer cases ($y=3.34+0.063x$, $r=0.20$NS, df=28); controls ($y=3.95+0.044x$, $r=0.16$NS, df=286).

the regression of concentration on age, (and the 95% reference ranges) for the normal controls. It is interesting that there was a significant increase of vitamin A and RBP values with increasing age. A similar trend occurred with vitamin E but the statistical analysis was short of formal significance. The regression lines of vitamin A and RBP concentrations on age in the pre-cancer cases were very similar to those of the controls.

An excellent correlation was found between the serum vitamin A and RBP concentration in both the pre-cancer cases and the controls ($r=0.91$, $P<0.0001$), and this is in accordance with the findings of other workers (Smith & Goodman, 1976; Vahlquist *et al.*, 1978; Goodman, 1984). From the above results it is clear that measurement of serum RBP could be used to replace the more cumbersome HPLC method, in epidemiological studies.

### Discussion

Our results have not shown any significant relationship between serum concentrations of vitamin A, RBP and vitamin E, and subsequent development of breast cancer. A similar study of ovarian cancer also showed no differences as regards the two vitamins (Heinonen *et al.*, 1985). The finding of lower vitamin A values reported in men who develop lung cancer is supported by experimental work on induced tracheo-bronchial cancer (Saffiotti *et al.*, 1967; Cone & Nettesheim, 1973; Genta *et al.*, 1974). However, the majority of breast malignancies are adenocarcinomas in contrast to squamous and oat-cell lesions of the lung and this may explain the absence of any relationship with the retinoid environment.

Wald *et al.* (1984) found low vitamin E values in the pre-cancer cases whereas we did not, a surprising result because our subjects were drawn from the same population. It appears that the earlier results of Wald may have been due to serum samples from the cancer cases having been frozen and thawed more often than those from the controls (see Wald *et al.*, 1988). In the present experiment all frozen samples were intact and discarded after analysis.

Our study only includes 30 pre-cancer cases so that possibly some caution is needed in accepting the statistically negative findings. However, the ranking test used (Cuzick, 1985) is specially designed for statistical analysis in prospective studies where the number of cases is often limited, and controls are relatively plentiful. Up to 10 controls were used for comparison against each pre-cancer case in this particular study.

The increase in serum vitamin A concentrations with advancing age is an interesting finding. Although RBP and retinol normally circulate in the plasma at a 1:1 ratio, the turnover rate of the former is double that of vitamin A (Goodman, 1984). It is therefore possible that there is a reduction of RBP clearance in older women. Circulating RBP and vitamin A are influenced by natural and synthetic oestrogens which cause stimulation of hepatic RBP synthesis (Laurence & Sobel, 1953; Underwood, 1984) but this would not explain our results. Alternatively, the older women in this study may always have had a higher intake of $\beta$-carotene and vitamin E and the observed increases with age may reflect differences in dietary habits in the younger women.

Blood concentrations of retinol do not necessarily reflect tissue levels. Administration of high doses (50,000–200,000 IU) daily, resulted in plasma increases of retinyl esters but no change in vitamin A values thus suggesting deep tissue storage (Meyskens *et al.*, 1984). It is possible that blood levels of vitamin E may not be physiologically relevant since tissue fat is a major storage site (Kayden, 1983). The report that vitamin E is effective in the treatment of breast dysplasia (London *et al.*, 1981) although not confirmed (Ernster *et al.*, 1985), warrants an investigation of tissue levels in breast disease which we are now undertaking. Preliminary results suggest relatively large amounts of vitamin E, but very little retinol in peritumoral breast fat, both in benign and malignant conditions.

**References**

CHOW, F.I. & OMAYE, S.T. (1983). Use of antioxidants in the analysis of vitamins A and E in mammalian plasma by high performance liquid chromatography. *Lipids*, **18**, 837.

CONE, M.V. & NETTESHEIM, P. (1973). Effects of vitamin A on 3-methylcholanthrene-induced squamous metaplasias and early tumours in the respiratory tract of rats. *J. Natl Cancer Inst.*, **50**, 1599.

CUZICK, J. (1985). A method for analysing case-control studies with ordinal exposure variables. *Biometrics*, **41**, 609.

DRISKELL, W.J., BASHOR, M.M. & NEESE, J.W. (1985). Loss of vitamin A in long-term stored, frozen sera. *Clinica Chimica Acta*, **147**, 25.

ERNSTER, V.L., GOODSON, W.H., HURST, T.K., PETRAKIS, N.L., SICKLES, E.A. & MIIKE, R. (1985). Vitamin E and benign breast disease: A double blind clinical trial. *Surgery*, **97**, 490.

GENTA, V.M., KAUFMAN, D.G., HARRIS, C.C., SMITH, J.M., SPORN, M.B. & SAFFIOTTI, U. (1974). Vitamin A deficiency enhances binding of benzo-(a)-pyrene to tracheal epithelial DNA. *Nature*, **247**, 48.

GOFFERJE, H. (1978). Prealbumin and retinol-binding protein – highly selective parameters for the nutritional state in respect of protein. *Medical Laboratory*, **5**, 38.

GOODMAN, D.S. (1984). Plasma retinol binding protein. In *The Retinoids*, **Vol. 2**, Sporn, M.B. *et al.* (eds) p. 41. Academic Press Inc: London, UK and Orlando, FL.

HEINONEN, P.K., KOSINEN, T. & TUIMALA, R. (1985). Serum levels of vitamins A and E in women with ovarian cancer. *Arch. Gynecol.*, **237**, 37.

KARK, J.D., SMITH, A.H., SWITZER, B.R. & HAMES, C.G. (1981). Serum vitamin A (retinol) and cancer incidence in Evans County, Georgia. *J. Natl Cancer Inst.*, **66**, 7.

KAYDEN, H.J. (1983). Tocopherol content of adipose tissue from vitamin E deficient humans. In *Biology of Vitamin E, Ciba Foundation Symposium 101*, Porter, R. & Whelan, J. (eds) p. 70. Pitman Books Ltd: London, UK.

LAURENCE, P.A. & SOBEL, A.E. (1953). Changes in serum vitamin A level during the human menstrual cycle. *J. Clin. Endocrinol. Metab.*, **13**, 1192.

LONDON, R.S., SUNDARAM, G.S., SCHULTZ, M., NAIR, P.P. & GOLDSTEIN, P.J. (1981). Endocrine parameters and alpha-tocopherol therapy of patients with mammary dysplasia. *Cancer Res.*, **41**, 3811.

MENKES, M.S., COMSTOCK, G.W., VUILLEUMIER, J.P., HELSING, K.J., RIDER, A.A. & BROOKMEYER, R. (1986). Serum β-carotene, vitamins A and E, selenium and the risk of lung cancer. *N. Engl. J. Med.*, **315**, 1250.

MEYSKENS, F.L., MOON, T.E., ALBERTS, D.S. & RITENBAUGH, C. (1984). The risk of cancer and serum vitamins A and E and carotenoids. *N. Engl. J. Med.*, **311**, 121.

RUSSELL, M.J., THOMAS, B.S. & WELLOCK, E. (1986). Simultaneous assay of serum vitamin A and vitamin E by high performance liquid chromatography using time-switched UV and fluorimetric detectors. *HRC & CC*, **9**, 281.

SAFFIOTTI, U., MONTESANO, R., SELLAKUMAR, A.R. & BORG, S.A. (1967). Experimental cancer of the lung. Inhibition by vitamin A of the induction of tracheo-bronchial squamous metaplasia and squamous cell tumours. *Cancer*, **20**, 857.

SMITH, F.R. & GOODMAN, D.S. (1976). Vitamin A transport in human vitamin A toxicity. *N. Engl. J. Med.*, **294**, 805.

STAHELIN, H.B., BUESS, E., ROSEL, F., WIDMER, L.K. & BRUBACHER, G. (1982). Vitamin A, cardiovascular risk factors and mortality. *Lancet*, **i**, 394.

TYLER, H. & DICKINSON, J.W.T. (1984). Determination of serum retinol in cancer studies. *Eur. J. Cancer Clin. Oncol.*, **20**, 1205.

UNDERWOOD, B.A. (1984). Vitamin A in animal and human nutrition. In *The Retinoids*, **Vol. 1**, Sporn, M.B. *et al.* (eds) p. 282. Academic Press Inc: London, UK and Orlando, FL.

VAHLQUIST, A., SJOLUND, K., NORDEN, A., PETERSON, P.A., STIGMAR, G. & JOHANSSON, B. (1978). Plasma vitamin A transport and visual dark adsorption in diseases of the intestine and liver. *Scand. J. Clin. Lab. Invest.*, **38**, 301.

WALD, N.J., BOREHAM, J. & BAILEY, A. (1980). Low serum vitamin A and subsequent risk of cancer. Preliminary results of a prospective study. *Lancet*, **ii**, 813.

WALD, N.J., BOREHAM, J., HAYWOOD, J.L. & BULBROOK, R.D. (1984). Plasma retinol, β-carotene and vitamin E levels in relation to the future risk of breast cancer. *Br. J. Cancer Clin. Oncol.*, **49**, 321.

WALD, N.J., BOREHAM, J. & BAILEY, A. (1986). Serum retinol and subsequent risk of cancer. *Br. J. Cancer*, **54**, 957.

WALD, N.J., NICOLAIDES-BOUMAN, A. & HUDSON, G.A. (1988). Plasma retinol, beta-carotene and vitamin E levels in relation to the future risk of breast cancer. *Br. J. Cancer*, **57**, 235.

WILLET, W.C., POLK, B.F., UNDERWOOD, B.A. & 6 others (1984). Relation of serum vitamins A and E and carotenoids to the risk of cancer. *N. Engl. J. Med.*, **310**, 430.